# Structural and functional brain alterations associated with cancer‐associated cognitive decline in gastric cancer patients: A preliminary longitudinal neuroimaging study

**DOI:** 10.1002/brb3.2437

**Published:** 2021-11-25

**Authors:** Jaeun Ahn, DeokJong Lee, Young‐Chul Jung, Kyung Ran Kim

**Affiliations:** ^1^ Department of Psychiatry Yonsei University College of Medicine Seoul South Korea; ^2^ Institute of Behavioral Science in Medicine Yonsei University College of Medicine Seoul South Korea; ^3^ Department of Psychiatry Ilsan Hospital, National Health Insurance Corporation Goyang Republic of Korea; ^4^ Department of Psychiatry Yongin Severance Hospital Yonsei University College of Medicine Yongin South Korea

**Keywords:** cancer‐associated cognitive decline, chemotherapy, functional magnetic resonance imaging, gastric cancer, hippocampus, magnetic resonance imaging, neuroimaging

## Abstract

**Objective:**

Despite the clinical significance of cancer‐associated cognitive decline (CACD), no longitudinal study has evaluated CACD in gastric cancer patients. This preliminary study explored structural and functional neural changes of CACD in gastric cancer patients focusing on the effects of chemotherapy.

**Methods:**

13 gastric cancer patients who received adjuvant chemotherapy (CTx+ group), 9 gastric cancer patients who did not receive adjuvant chemotherapy (CTx‐ group), and 10 healthy controls (HCs) were enrolled in this study. We performed self‐report questionnaires, neurocognitive tests, voxel‐based morphometry (VBM), and resting‐state functional magnetic resonance imaging (rsfMRI) analyses before and 3 months after chemotherapy.

**Results:**

Compared to the CTx‐ group, the CTx+ group exhibited statistically significant decrease in attention and executive function over time and dysfunction in delayed recognition performance. The results of the rsfMRI analysis showed a significant group‐by‐time interaction in the left hippocampus–anterior thalamus. However, no significant structural change was observed in the VBM analysis.

**Conclusion:**

To the best of our knowledge, this is the first longitudinal neuroimaging study on CACD in gastric cancer patients. Based on the results of our preliminary study, we suggest that the neuropathological processes and clinical presentation of CACD in gastric cancer patients is similar to those of patients associated with age‐related neurodegenerative disorders.

## Graphical abstract

This is the first longitudinal neuroimaging study on chemobrain in male gastric cancer patients. The findings indicate that the clinical presentation and neuropathological processes affecting the hippocampus are similar to those observed in age‐related neurodegenerative disorders.

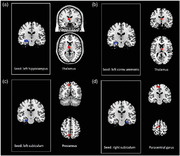

## 1 INTRODUCTION

In adults, cancer‐associated cognitive decline—also referred to as CACD—for non‐central nervous system types of cancers is the most feared problems among cancer survivors, since it is directly related to social and occupational dysfunction and decreased quality of life (Ahles et al., 2012). Research on the concept of CACD was initiated from pharmacotoxicology concept as a side effect of chemotherapy, but recently, the concept of CACD has progressed multidimensionally as CACD is related to complex interactions of various factors such as biology of aging, biology of cancer, cancer treatments, and other factors that confer risk for post‐treatment cognitive decline (Ahles & Root, 2018).

Given its clinical significance, there has been increasing number of neuroimaging researches on the CACD phenomenon (Li & Caeyenberghs, 2018). Previous voxel‐based morphometry (VBM) studies showed reduced gray matter (GM) in breast cancer patients in frontal, temporal, parietal, and occipital regions 1 month following chemotherapy compared to baseline (Lepage et al., 2014; McDonald et al., 2013). Functional magnetic resonance imaging (fMRI) studies also suggested default mode (Kesler, 2014), executive mode (Wang et al., 2016) network abnormalities in CACD. However, despite growing evidence from neuroimaging researches, neural correlates for CACD are not defined (Ahles & Root, 2018).

Most studies on CACD as of now were limited to breast cancer patients due to frequent occurrence and high survival rates in this population (Jung et al., 2019; Li & Caeyenberghs, 2018; Oh et al., 2016). However, we decided to focus our study on CACD in gastric cancer patients because we wanted to direct our attention to chemotherapy among various other factors affecting CACD since previous studies have speculated that chemotherapy may affect certain brain regions and cognitive aging (Ahles et al. 2012; Mandelblatt et al., 2014). First, we aimed to reduce the effect of biology of aging on CACD as gastric cancer has high incidence rate and survival rate is high for middle aged men in South Korea. In South Korea, gastric cancer is the most prevalent cancer in males aged 35 to 64, while breast cancer is the most common in women aged 35 to 64 (Hong et al., 2020). Although the 5‐year survival rate of gastric cancer (76.5% from 2013 to 2017) is lower than that of breast cancer (93.2% from 2013 to 2017), 5‐year survival rate of gastric cancer has jumped from 43.9% in 1993 to 76.5% in 2017 (Hong et al., 2020). Second, we aimed to reduce the effect of brain metastases on CACD since brain metastases in gastric cancer are exceedingly rare (<1%) (Go et al., 2011), while brain metastases from breast cancer are second to most common after lung cancer (13–19%) (Nayak et al., 2012). In gastric cancer, fluorouracil, oxaliplatin, and capecitabine are recommended as chemotherapeutic agents (Network, 2021), while in breast cancer, 5‐fluorouracil, capecitabine, doxorubicin, paclitaxel, cyclophosphamide, etc., are frequently recommended as chemotherapeutic agents (Network NCC, 2021). Considering these points, we judged that gastric cancer patients would be a good target group to study CACD phenomenon with the focus on chemotherapy.

The purpose of this preliminary study was to identify alterations in gray matter and functional connectivity related to CACD in gastric cancer patients. Neuropsychological tests were performed to assess attention, concentration, memory, and executive functions in patients with (CTx+) and without (CTx‐) adjuvant chemotherapy at baseline and follow‐up. The neuroimaging analyses focused on areas where differences between baseline and follow‐up were associated with changes in neurocognitive assessment results. We attempted to identify whether GM changes occur following chemotherapy through VBM analysis. Seed‐based resting‐state functional connectivity analyses were performed to investigate neural network changes. To the best of our knowledge, this longitudinal research is the first study undertaken to identify underlying structural and functional changes associated with CACD in patients with gastric cancer, using neuroimaging techniques with a focus on the hippocampus.

## 2 METHODS

### 2.1 Participants

This prospective study was approved by the Institutional Review Board of Severance Hospital (4‐2012‐0677), and informed written consents were obtained from all subjects before each procedure. Male gastric cancer patients between ages 40 and 60 who underwent total or partial (distal or subtotal) gastrectomy were enrolled in this study. We recruited only male gastric patients due to the higher gastric cancer prevalence rate in males in Korea, which is twice as high than that in women (Jung et al., 2019) and for sample homogeneity. Candidates were divided into two groups: patients with scheduled adjuvant chemotherapy (CTx+) and patients who did not need adjuvant chemotherapy (CTx‐ group). The CTx+ group received XELOX (capecitabine and oxaliplatin) or TS‐1 (Tegafur, gimeracil, and oteracil potassium) chemotherapeutic regimens. The TS‐1 regimen is a regimen with a 1‐year schedule that begins 6 weeks after surgery and involves cycles of a 4‐week period of chemotherapy followed by a 2‐week break. XELOX is an anticancer medication that follows a schedule of eight cycles of treatment at 3‐week intervals. In addition, an ag‐ and sex matched healthy control (HC) group without cognitive impairment or active neurological disorders was also recruited. Potential participants who had a history of (1) other malignancies; (2) metastatic malignancies; (3) any neurologic condition that could impair cognitive function (neurodegenerative disease, stroke, brain injury, etc.); (4) alcohol, nicotine, caffeine, or other drug dependence or addiction; or (5) Axis I psychiatric disorder were excluded.

Initial baseline assessments were performed on the CTx+ group (*n* = 19; age: 49.2 ± 5.5), CTx‐ group (*n* = 14; age: 49.2 ± 6.8), and HC group (*n* = 10; age: 51.5 ± 7.0). Baseline assessments were performed using self‐report questionnaires, neurocognitive assessments, and MRI scanning on the same day, after gastric cancer surgery and before adjuvant chemotherapy. In the CTx+ group, follow‐up assessment was performed approximately 3 months after the subjects underwent adjuvant chemotherapy. The CTx‐ group was assessed at a matched interval.

### 2.2 Self‐report questionnaires and neurocognitive assessment

The self‐report questionnaires included cognitive failure questionnaire (CFQ) (Broadbent et al., 1982) to assess subjective cognitive decline, the Beck depression inventory (BDI) (Beck et al., 1996) to assess depressive symptoms, and the Beck anxiety inventory (BAI) (Steer & Beck, 1997) to assess anxiety symptoms. All subjects completed a structured clinical interview, and an assessment of major psychiatric illness was performed based on the Diagnostic and Statistical Manual of Mental Disorders, Fourth Edition (First et al., 1995). Four cognitive domains were assessed using a set of neurocognitive tests: (1) performance and verbal intelligence (Korean version of Wechsler Adult Intelligence Scale [K‐WAIS] performance and verbal subtests), (2) memory (Rey‐Kim Memory Test), (3) attention (K‐WAIS digit span and spatial span subtests), and (4) executive function (Stroop test). Scores for the neurocognitive tests are expressed as age‐corrected scaled scores (AgeSS), standardized scores (SS), or percentile ranks for raw scores.

### 2.3 Functional connectivity analysis

All participants underwent rsfMRI in a 3 T MR scanner (Tim Trio, Siemens Healthcare, Erlangen, Germany) equipped with a 16‐channel head coil. rsfMRI data were acquired using gradient echo‐planar pulse imaging (EPI). For each subject, 150 axial volume scans were obtained with the following parameters: TR  =  3000 ms, TE  =  30 ms, FOV  =  192 × 192 mm^2^, voxel size  =  3 × 3 × 3 mm^3^, and slice number  =  50 (interleaved). During the acquisition of fMRI data, subjects were directed to look at a white cross in the center of a black background for 7 min 30 s without any cognitive, lingual, or motor activities. Vacuum‐molded cushions and soft pads were used to support the head and minimize head movement.

Spatial preprocessing and statistical analyses of functional images were performed using SPM12 (Welcome Department of Imaging Neuroscience, London, UK). To analyze functional connectivity based on resting‐state fMRI data, motion artifacts were assessed in individual subjects by visually inspecting realignment parameter estimations to confirm that there were no abrupt head motions and that the maximum head motion in each axis was < 3 mm. Functional images were realigned and registered to structural images for each subject. The anatomical volume was segmented into GM, white matter, and cerebrospinal fluid. The GM image was used to determine the normalization parameters onto the standard MNI GM template provided with SPM12. The spatial parameters were then applied to the realigned functional volumes that were finally resampled to voxels of 2 × 2 × 2 mm^3^ and smoothed with an 8‐mm full‐width at half‐maximum kernel.

Cortical network assessment was performed using a region of interest (ROI) seed‐based correlation approach. Connectivity analysis was conducted with the “conn” toolbox implemented in SPM12 (http://www.fil.ion.ucl.ac.uk/spm/ext). Seed areas were chosen using the automated anatomical labeling (AAL) template (www.fil.ion.ucl.ac.uk/spm/ext/) (Tzourio‐Mazoyer et al., 2002). Based on the between‐group differences over time observed with the neurocognitive testing, the following ROIs were selected: the superior parietal lobe, which is associated with spatial span (Berryhill & Olson, 2008); the bilateral hippocampus, which is associated with recognition (Pospisil et al., 2015; Zhou et al., 2010); and the bilateral superior frontal gyri (SFG), which is associated with executive function. A growing body of evidence indicates that the hippocampus is divided into several subfields with specific cognitive functions (Mueller et al., 2010). In particular, it has been argued that neurodegenerative disorder patients show a sequential pattern of changes starting within the entorhinal and transentorhinal areas and moving to cornu ammonis area 1 (CA1), subiculum, and eventually other subfields. To study the acute neurotoxic effects of chemotherapy, we conducted further analysis by classifying six hippocampal subfields and setting them as seed regions. ROIs for the subfields of the bilateral hippocampus (consisting of cornu ammonis [CA, including CA1, CA2, and CA3 subfields]; dentate gyrus [DG] including the fascia dentata and CA4 subfield; and subiculum [SB] including the prosubiculum, subiculum proper, presubiculum, and parasubiculum) were obtained from the maximum probability map (MPM) (Eickhoff et al., 2005). These were defined using the Anatomy toolbox v22c implemented in SPM12 (www.fil.ion.ucl.ac.uk/spm). The waveform from each brain voxel was temporally filtered by means of a bandpass filter (0.008 Hz < *f* < .09 Hz) to adjust for low‐frequency drift and high‐frequency noise effects. Linear regression analysis was conducted to remove signals from the ventricular area and white matter (Whitfield‐Gabrieli & Nieto‐Castanon, 2012). Movement parameters were added as first‐level covariates. To estimate the functional connectivity strength, correlation coefficients were computed and converted to *z*‐values using Fisher's r‐to‐z transformation.

### 2.4 Voxel‐based morphometry analysis

The three‐dimensional structural MRI data were acquired through a T1‐weighted spoiled gradient echo sequence (repetition time  =  1900 ms; echo time  =  2.52 ms; FOV  =  256 × 256 mm^2^; voxel size  =  1 × 1 × 1 mm^3^; flip angle 9°; slice number  =  176; and total acquisition time: 4 min 26 s). Structural brain images were analyzed with MATLAB version 9.3 (R2020a; MathWorks, Natick, MA, USA) and SPM12 (Wellcome Department of Imaging Neuroscience, UK). All preprocessing steps were conducted in accordance with a standardized procedure (Ashburner, 2007). First, the structural images were aligned along the anterior–posterior commissure line and positioned so that the anterior commissure matched the origin. Afterward, the images were segmented into GM, white matter, and cerebrospinal fluid probability maps using a Bayesian image segmentation algorithm. Brain tissue probability maps for each subject were then used for intersubject alignment. In this study, we applied diffeomorphic anatomical registration using an exponentiated Lie algebra algorithm (DARTEL; (Ashburner, 2007)). The DARTEL has been suggested to enhance intersubject alignment accuracy by modeling the shape of each brain using a host of parameters. DARTEL processing involves generating the flow fields that parameterize deformations and creating templates for all subjects. After the final study‐specific template was created, GM images for each subject were warped to the study‐specific template and then normalized into standard Montreal Neurological Institute (MNI) space. The volumes were resampled to a 1.5 × 1.5 × 1.5 mm^3^ voxel size. This spatial normalization step included Jacobian modulation to preserve regional volume data. Finally, the DARTEL‐warped, normalized, and modulated GM images were smoothed using an 8‐mm full‐width at half maximum kernel.

### 2.5 Statistical analysis

Baseline demographic characteristics, including age, years of education, and self‐report questionnaire data, were compared between gastric cancer patients with one‐way analyses of variance (ANOVAs). For the neurocognitive assessments, we compared changes in performance using results from baseline and 3 months after adjuvant chemotherapy with a repeated measures ANOVA for a significance level of *p* = .05 between the CTx+ and CTx‐ groups.

Between‐group and within‐group longitudinal comparisons and group‐by‐time interaction analyses were conducted to compare functional connectivity strengths. Statistical significance was set to an uncorrected *p*‐value height threshold of 0.001 and *k* > 100 (Bédard et al., 2014) as the extent threshold for the whole brain.

Additionally, between‐group and within‐group longitudinal comparisons and group‐by‐time interaction analyses were conducted for voxelwise comparisons of GMV between the CTx+ group and CTx‐ group. Age and intracranial volume were entered as covariates because of their known influence on GMV (Pell et al., 2008). Intracranial volume for each subject was calculated by the sum of gray matter, white matter, and cerebrospinal fluid volume. Whole‐brain analyses were conducted to locate clusters showing group differences in GMV. A voxel wise cluster‐defining threshold of uncorrected *p* < .001 was applied. All statistical analyses were conducted with SPSS 25.0 software (IBM, Armonk, NY, USA).

## 3 RESULTS

### 3.1 Demographic characteristics

**FIGURE 1:**
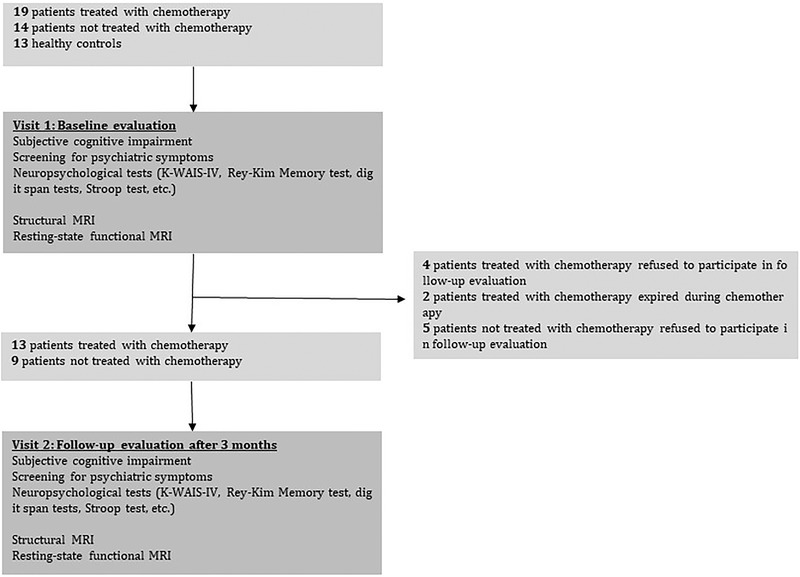
Study flowchart. K‐WAIS‐IV, Korean Wechsler Adult Intelligence Scale‐IV; MRI, magnetic resonance imaging

We initially enrolled 19 gastric cancer patients who underwent total or subtotal gastrectomy and were candidates for adjuvant chemotherapy and 14 patients who underwent total or partial gastrectomy (distal gastrectomy, subtotal gastrectomy) but did not need adjuvant chemotherapy. All participants did not receive radiotherapy or other treatments except for surgical treatment and adjuvant chemotherapy. Of the 19 patients in the original CTx+ group, 6 were excluded (2 patients expired during chemotherapy and 4 refused follow‐up assessment after chemotherapy). Five patients who were enrolled in the CTx‐ group were excluded because they refused follow‐up assessment. Thirteen age‐matched healthy males were recruited as control subjects. Among them, two HC participants were excluded since they showed impairment on the neurocognitive tests. One HC participant was excluded due to failure to complete the multimodal neuroimaging studies. No participant was excluded due to excessive head movement during fMRI scanning. Therefore, the final sample size for the longitudinal analysis was 38, and all subjects had completed formal education. The breakdown of sample sizes was as follows: 13 in the CTx+ group, 9 in the CTx‐ group, and 10 in the HC group (Figure 1).

With regard to cancer staging, the CTx+ group consisted of one stage I patient, three stage II patients, eight stage III patients, and one stage IV patient who went through oxaliplatin+Xeloda or tegafur+gemaracil chemotherapy regimens. The CTx‐ group consisted of three stage I patients and six stage II patients. In the CTx+ group, follow‐ups were performed an average of 94.5 ± 10.5 days after surgery, and in the CTx‐ group, follow‐ups were performed an average of 92.6 ± 11.1 days after surgery. The implemented surgery type, protocol of adjuvant chemotherapy protocol, and cancer stages are summarized in Table 1. At baseline, there was no significant difference between the two groups in terms of age, duration of education, CFQ score, BDI score, or BAI score (Table 2, 3).

**TABLE 1:** Clinical characteristics of gastric cancer patients

	**CTx+ (*n* = 13)**	**CTx‐ (*n* = 9)**
**Gastrectomy type**		
Total	4	0
Subtotal	8	2
Distal	1	7
**Cancer stage**		
I	1	3
II	3	6
III	8	0
IV	1	0
**Adjuvant chemotherapy protocol**		
XELOX	7	N/A
TS‐1	6	N/A

*Notes*: CTx+, patients treated with chemotherapy; CTx‐, patients not treated with chemotherapy; XELOX, oxaliplatin+Xeloda; TS‐1, tegafur+gemaracil+oteracil potassium.

**TABLE 2:** Demographic and clinical characteristics of gastric cancer patients and controls at baseline

	**CTx+ (*n* = 13) Mean (SD)**	**CTx‐ (*n* = 9) Mean (SD)**	**HCs (*n* = 10) Mean (SD)**	F	*p*
Age (years)	49.2 (5.5)	49.2 (6.8)	51.5 (7.0)	0.472	0.628
Years of education	13.1 (3.2)	13.3 (2.0)	12.7 (1.6)	0.189	0.828
BDI	9.8 (7.4)	8.2 (6.8)	10.7 (6.9)	0.366	0.696
BAI	6.0 (6.6)	6.9 (5.4)	6.2 (5.9)	0.075	0.928
CFQ	9.5 (9.7)	16.8 (11.0)	19.8 (16.3)	2.248	0.121

*Notes*: Data are means ± standard deviations. CTx+: patients treated with chemotherapy; CTx‐: patients not treated with chemotherapy.

Abbreviations: BAI, Beck Anxiety Inventory; BDI, Beck Depression Inventory; CFQ, Cognitive Failure Questionnaire; HCs, healthy controls.

**TABLE 3:** Neurocognitive assessments before and after chemotherapy

**Domain/test**	**CTx+ (*n* = 13)**			**CTx‐ (*n* = 9)**			***Repeated measures ANOVA: group‐by‐time interaction***
	Baseline *Mean (SD)*	3‐month follow‐up *Mean (SD)*	*p* ^†^	Baseline *Mean (SD)*	3‐month follow‐up *Mean (SD)*	***p*** ^†^	
							*p*
**Attention and concentration**							
Digit span forward	10.8 (3.0)	10.2 (3.2)	.407	9.2 (2.3)	9.9 (1.9)	.322	.863
Digit span backward	11.3 (3.3)	10.9 (3.5)	.539	10.6 (2.1)	10.3 (2.3)	.520	.419
Digit span total	11.1 (3.1)	11.2 (3.0)	.711	9.6 (2.1)	9.9 (1.9)	.576	.491
Spatial span forward (%)	35.8 (33.9)	41.5 (33.2)	.360	27.1 (32.7)	22.6 (25.6)	.474	.155
Spatial span backward (%)	79.5 (14.5)	72.4 (20.3)	.260	69.1 (26.6)	71.3 (28.6)	.776	.020*
**Memory**							
Rey–Kim memory quotient	102.5 ± 14.1	108.2 ± 10.3	.131	108.7 ± 12.0	110.6 ± 11.7	.326	.072
AVLT‐sum	10.4 ± 3.0	11.6 ± 1.8	.220	12.1 ± 3.6	11.5 ± 2.6	.310	.632
AVLT‐delayed recall	9.5 ± 2.6	10.2 ± 2.1	.248	11.0 ± 2.2	11.9 ± 2.0	.159	.069
AVLT‐delayed recognition	9.6 ± 3.7	10.4 ± 3.5	.117	11.0 ± 2.7	12.8 ± 2.5	.068	.011*
KCFT copy	14.7 ± 1.8	14.3 ± 2.1	.910	14.3 ± 5.1	14.6 ± 2.0	.563	.927
KCFT immediate recall	13.1 ± 3.7	14.2 ± 2.0	> .999	14.3 ± 1.9	14.3 ± 1.8	.145	.305
KCFT delayed recall	12.9 ± 3.6	14.5 ± 1.9	.674	13.7 ± 2.3	14.0 ± 2.0	.116	.150
**Executive Function**							
STROOP (%)	56.0 ± 41.1	48.2 ± 37.6	.097	56.2 ± 37.3	60.4 ± 34.2	.631	< .001*

*Notes*: CTx+: gastric cancer patients treated with chemotherapy; CTx‐: gastric cancer patients not treated with chemotherapy;

*p* ^†^: results of paired t‐test comparing before to 3 months after adjuvant chemotherapy in each CTx+ and CTx‐ group.

Abbreviations: AVLT, auditory verbal learning test; KCFT, Korean complex figure test.

### 3.2 Neurocognitive results

We compared the neurocognitive results of the CTx+ and CTx‐ groups (Table 2). Repeated measures ANOVA demonstrated significant between‐group differences over time in the spatial span backward test (*p* = .020), auditory verbal learning test (delayed recognition, *p* = .001), and Stroop test (*p* < .001).

### 3.3 Neuroimaging analysis

#### 3.3.1 VBM analysis

Comparing the CTx+ and CTx‐ groups, the between‐group and within‐group differences and the group‐by‐time interaction were not significant.

#### 3.3.2 Functional connectivity analysis

**FIGURE 2:**
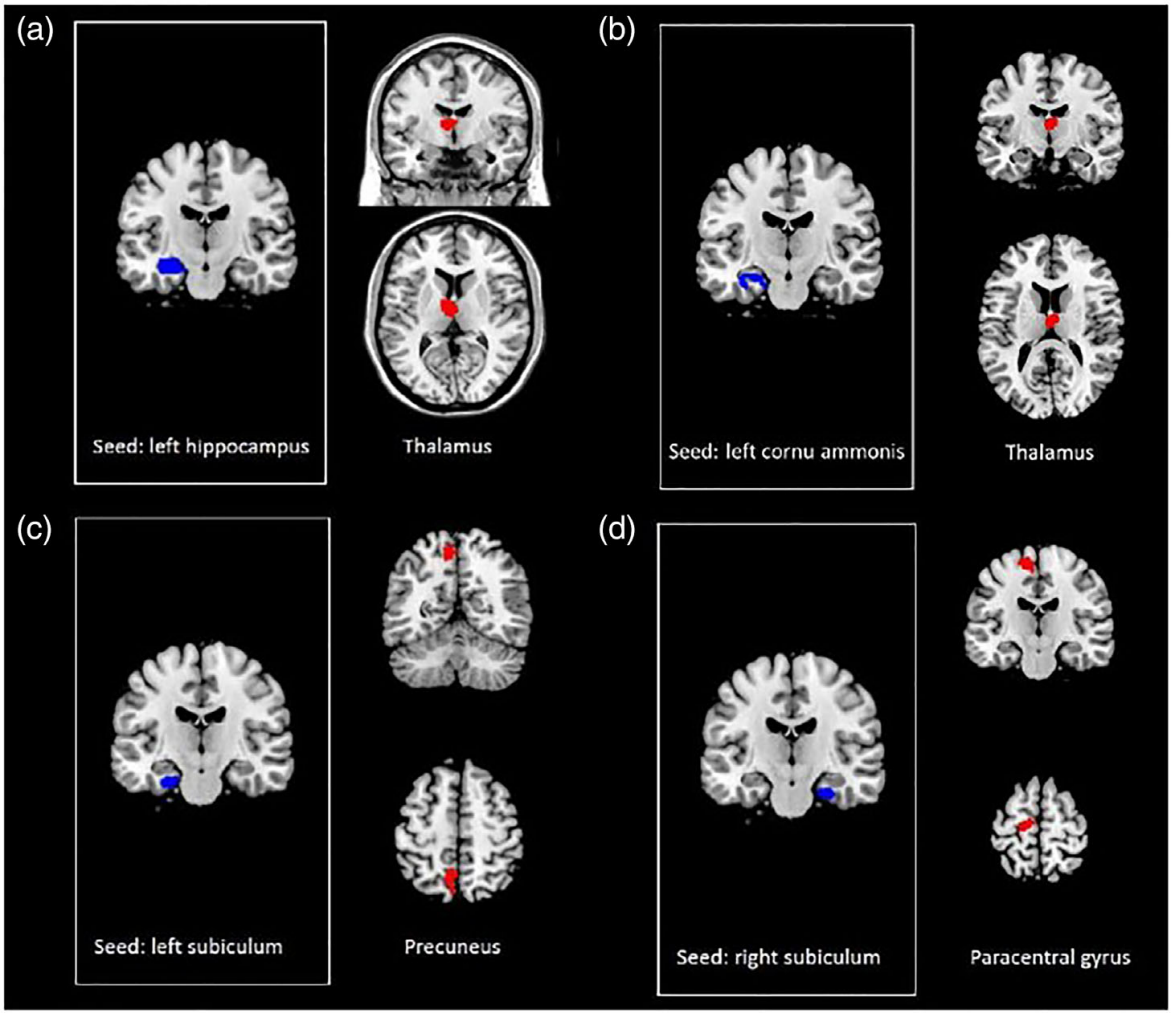
Brain regions showing significant group‐by‐time interactions in the hippocampus and hippocampal subfield‐based functional connectivity analyses. Statistical inferences were thresholded using an uncorrected *p* < .001, k_E_ > 100 voxels for the whole brain

When seeding from the bilateral hippocampus, there were no significant group differences in functional connectivity between the CTx+ and CTx‐ groups. However, functional connectivity analysis showed a significant group‐by‐time interaction in the anterior thalamus (Table 4; Figure 2). Post hoc analyses using ROIs of the hippocampal subfields showed significant group‐by‐time interactions with left CA—anterior thalamus functional connectivity, left subiculum—precuneus functional connectivity, and right subiculum—paracentral gyrus functional connectivity.

**TABLE 4:** Brain regions showing significant group‐by‐time interactions in the hippocampus‐ and hippocampal subfield‐based functional connectivity analysis

**Region**	**Side**	**BA**	**K**	***T*_max_**	**Coordinates**		
					**x**	**y**	**z**
**Functional connectivity with the left hippocampus**							
Thalamus, anterior nuclei	Left		305	4.82	−4	−12	12
**Functional connectivity with the left cornu ammonis**							
Thalamus, anterior nuclei	Right		228	4.92	2	−10	14
**Functional connectivity with the left subiculum**							
Precuneus	Left	7	122	5.14	−6	−58	56
**Functional connectivity with the right subiculum**							
Paracentral gyrus	Left	4	122	4.49	−12	−20	66

For all ROI‐based functional connectivity results, with the exception of the hippocampus‐based analyses, there were no between‐group or within‐group significant differences or significant group‐by‐time interactions.

## 4 DISCUSSION

To our knowledge, this is the first prospective, longitudinal neuroimaging study on chemotherapy‐induced cognitive impairment in gastric cancer patients. The results revealed that compared to the patients in the CTx‐ group, the patients in the CTx+ group had dysfunction in attention, memory, and executive function 3 months after chemotherapy. rsfMRI analyses that set the hippocampus—a key region for memory function—as the seed showed altered left hippocampus—anterior thalamus connectivity in the context of CACD. Moreover, additional hippocampal subfield analyses identified altered left CA—anterior thalamus connectivity. These findings provide evidence of neuropathological changes related to CACD with qualitatively different neural changes in the CTx+ group compared to the CTx‐ group; however, VBM analysis did not show any structural changes.

Objective neurocognitive tests showed that adjuvant chemotherapy in patients with gastric cancer may lead to dysfunction in attention, memory, and executive function. Previous studies have yielded inconsistent objective neurocognitive test results regarding subjective cognitive difficulties after chemotherapy due to variability in study design (Ahles & Root, 2018). Moreover, there are still many arguments regarding which cognitive function domains are affected by CACD (Li & Caeyenberghs, 2018). The standardized neuropsychological test commonly used was developed to determine lesion location and impairment in patients with overt neurological injuries and illnesses. In contrast, the cognitive dysfunction in CACD is relatively subtle, and measurement error in traditional objective neuropsychological test alone could obscure true changes (Ahles & Root, 2018). Furthermore, objective neurocognitive tests might be affected by expectation effects and test‐retest effects due to longitudinal design (Li & Caeyenberghs, 2018). Nevertheless, we observed impairments in attention, memory, and executive function in objective neurocognitive tests in gastric cancer patients after chemotherapy, although we enrolled middle‐aged subjects with normal cognitive function who had completed formal education to exclude factors affecting CACD such as aging and cognitive reserve (Ahles & Root, 2018). These findings suggest that it is necessary to identify CACD symptoms early and accurately in gastric cancer patients, and it is also important to perform appropriate therapeutic intervention such as cognitive rehabilitation and pharmacological treatment.

In our study, GM alterations were not observed in CACD. Considering that changes in functional connectivity precede structural brain changes, it can be assumed that the reason for the lack of GM volume changes in CACD is because only short‐term acute impacts were evaluated in this study. Previous meta‐analysis of GM abnormalities in the CACD suggested that cognitive impairment in cancer patients after chemotherapy varies over time (Niu et al., 2020). It was suggested that early brain functional alterations may be related to the direct response of neurons to chemotherapy, while long‐term brain functional alterations may be related to persistent brain structural alterations, which seem to gradually recover over the years.

Through functional connectivity analyses, we were able to identify qualitatively different alterations in left hippocampus—anterior thalamus functional connectivity in the CTx+ group compared to the CTx‐ group. Although the neuropathological mechanisms underlying CACD remains unclear, there is evidence suggesting that the hippocampus may be a vulnerable area in CACD (Feng et al., 2019; Peukert et al., 2020). In various rodent studies, CACD was related to impaired neurogenesis in the hippocampus (ELBeltagy et al., 2010; Lyons et al., 2012), neuroinflammation (Acharya et al., 2015; Christie et al., 2012), oxidative stress, mitochondrial dysfunction (Oz et al., 2015; Ramalingayya et al., 2017), and structural damage to neurons. The hippocampus is also a key area in memory formation, learning (Squire et al., 1992), spatial processing (Richard et al., 2013), memory recognition, and prospective memory processing (Gordon et al., 2011). Moreover, the hippocampus is a key domain in cognitive function that is also involved in the pathogeneses of neurodegenerative disorders (Braak & Braak, 1997; Buckner et al., 2008). Additionally, the anterior thalamus is a pivotal area in memory and cognition, and hippocampal—anterior thalamic interconnections play vital roles in human memory and cognition (Aggleton et al., 2010). In addition, the change in the connectivity between the hippocampal CA (Atienza et al., 2011) and thalamus (which is also known to be initially affected in neurodegenerative disorders such as AD) suggests that neural changes due to CACD are similar to those in the early stages of age‐related neurodegenerative disorders. Therefore, future studies based on long‐term follow‐up should be carried out to determine how resting‐state functional connectivity changes progress in the context of CACD.

### 4.1 Study limitations

There are several limitations in this study. First, the study had a small sample size due to difficulty in recruiting cancer patients as participants. This might be why we found no significant correlations between neuroimaging findings and neurocognitive test results, although we did identify changes in brain regions associated with objective neurocognitive assessments. Second, the follow‐up period was insufficient. Assessing larger study populations for longer times could clarify how cognitive impairment and neural changes develop, which would be more helpful for understanding the underlying mechanism of CACD. Third, average cancer stage of CTx+ group was higher than that of CTx‐ group in this study, mainly because adjuvant chemotherapy was required in patients with higher cancer stages. Although no specific mechanism has been identified yet, there is perspective that cancer biology itself can affect CACD (Ahles & Root, 2018). In the future, it is needed to conduct the study of CACD which controls the difference of cancer stage. Lastly, the repetition time for the rs‐fMRI scans was long. In future studies, a shorter repetition time can improve the statistical power of the results of the study.

### 4.2 Clinical implications

To our knowledge, this is the first prospective longitudinal neuroimaging study of CACD in gastric cancer patients. To control factors leading to CACD other than chemotherapy, we attempted to select subjects who are middle‐aged with normal cognitive functions who had completed formal education, have no history of treatment other than surgery and chemotherapy, have no brain metastasis, no history of psychiatric disorder, and are not significantly affected by psychological effects such as depression and anxiety after surgery.

Results of this study show that adjuvant chemotherapy in gastric cancer patients might affect declines in attention, memory, and executive function that are accompanied by underlying neural changes. Although the biological underpinning of CACD have not been clearly or universally explained, previous results supported the hypothesis that the pathological processes and clinical presentation of CACD are ultimately similar to age‐related neurodegenerative disorders (Ahles et al., 2010; Li & Caeyenberghs, 2018). Our findings that altered functional activation in the hippocampus resonate with previous studies showing hippocampal changes of CACD in other cancer populations. In the future, longitudinal studies should be carried out in a large, homogenous sample of patients. Such work will deepen our understanding of the mechanisms underlying CACD and will enable more effective therapeutic interventions.

## 5 CONCLUSIONS

This is the first prospective longitudinal neuroimaging study on CACD in male gastric cancer patients. The findings indicate that the clinical presentation and neuropathological processes affecting the hippocampus of CACD in male gastric cancer patients may be similar to those observed in age‐related neurodegenerative disorders.

## CONFLICT OF INTEREST

All authors declare no conflicts of interest.

## FUNDING

This research was supported by a health fellowship foundation. Role of the funding source: The funding source of this study had no role in the design of this study and did not have any role during its execution, analyses, interpretation of the data, or decision to submit results.

### PEER REVIEW

The peer review history for this article is available at https://publons.com/publon/10.1002/brb3.2437.

## Data Availability

The data that support the findings of this study are available on request from the corresponding author. The data are not publicly available due to privacy or ethical restrictions.
